# The DNA topoisomerase II inhibitor amsacrine as a novel candidate adjuvant in a model of glaucoma filtration surgery

**DOI:** 10.1038/s41598-019-55365-7

**Published:** 2019-12-17

**Authors:** Kotaro Yamamoto, Taiki Kokubun, Kota Sato, Takahiro Akaishi, Atsushi Shimazaki, Masatsugu Nakamura, Yukihiro Shiga, Satoru Tsuda, Kazuko Omodaka, Hideyuki Saya, Toru Nakazawa

**Affiliations:** 1 Department of Ophthalmology, Tohoku University Graduate School of Medicine, Sendai, Miyagi 980-8574 Japan; 2 Department of Collaborative Program for Ophthalmic Drug Discovery, Tohoku University Graduate School of Medicine, Sendai, Miyagi 980-8574 Japan; 3 Research and Development Division, Santen Pharmaceutical Co. Ltd., Ikoma, Nara 630-0101 Japan; 4 Division of Gene Regulation, Institute for Advanced Medical Research, School of Medicine, Keio University, 35 Shinanomachi, Shinjuku-ku, Tokyo 160-8582 Japan; 5 Department of Retinal Disease Control, Ophthalmology, Tohoku University Graduate School of Medicine, Sendai, Miyagi 980-8574 Japan; 6 Department of Advanced Ophthalmic Medicine, Tohoku University Graduate School of Medicine, Sendai, Miyagi 980-8574 Japan

**Keywords:** High-throughput screening, Experimental models of disease

## Abstract

Treatments for refractory glaucoma include trabeculectomy, in which a filtering bleb is created to reduce aqueous pressure. Mitomycin C (MMC) is often used as an adjuvant to reduce post-trabeculectomy bleb scarring and consequent failure. However, scarring sometimes still occurs. Thus, we searched for more effective trabeculectomy adjuvants with high-throughput screening (HTS) of a library of 1,165 off-patent drug compounds. This revealed that amsacrine (AMSA), a DNA topoisomerase II (TOP2) inhibitor, was the top candidate. Compared to MMC, rabbits that underwent trabeculectomy with 10% AMSA had lower IOP at 42, 56, and 70 days (*P* < 0.01 at all measurement points) and a higher bleb score at 28, 42, 56, and 70 days (*P* =  < 0.01, 0.04, 0.04, and < 0.01, respectively). Compared to saline, rabbits that received 1% AMSA also had lower IOP and better bleb score at all time points, without a sharp drop in IOP just after surgery (all *P* < 0.01). Both effects were milder than MMC at 7 days (*P* = 0.02 and <0.01, respectively). Thus, this study showed that HTS may help identify new, promising uses for off-patent drugs. Furthermore, trabeculectomy with AMSA at a suitable concentration may improve the prognosis after trabeculectomy compared to MMC.

## Introduction

Trabeculectomy is the most common surgical treatment for glaucoma, being used for glaucoma that is refractory to drug treatment^1,2^. This procedure was first described by Cairns in 1972, who reported that it allowed good intraocular pressure (IOP) control in about 70% of patients^3^. However, a successful outcome after trabeculectomy requires good postoperative wound healing. During healing, the excessive proliferation of fibroblasts in subconjunctival tissues, such as Tenon’s capsule, causes bleb failure^4,5^. Soon after the introduction of trabeculectomy, it was found that the intraoperative use of mitomycin C (MMC) hindered the growth of Tenon’s capsule fibroblasts (TCFs), thereby suppressing fibrosis in the surgical area and protecting the bleb^6^. The use of this adjuvant improved the success rate for trabeculectomy^6^, and became a standard part of the procedure^7,8^. However, despite the well-known benefits of MMC, the bleb can sometimes still fail, causing increased IOP to recur and necessitating additional treatment^9,10^.

Previous studies of bleb failure in trabeculectomy have often examined molecular dynamics in the anterior chamber^11–15^. The postoperative proliferation of TCFs is regulated by a complex signaling network that involves a large number of growth factors, cytokines and chemokines. Among these, the most important factors are the transforming growth factor-β (TGF-β) family^11,12^, connective tissue growth factor (CTGF)^13^, monocyte chemoattractant protein-1 (MCP-1)^14^, and platelet-derived growth factor (PDGF)^15^. However, the mechanism of bleb failure is still incompletely understood and it remains unknown which of these potential drug targets plays the most important role. Therefore, it is necessary to identify new, comprehensive, anti-proliferative compounds that can preserve bleb function more reliably than MMC, and can be chosen with consideration of the effects of differing bleb phenotypes.

Here, we attempted to discover novel, clinically useful drugs to serve as alternatives to MMC. We screened a drug library, comprising 1,165 off-patent drugs, evaluated the long-term effectiveness and safety profile of the identified candidate compounds in a rabbit model of trabeculectomy, and compared the results to those of MMC.

## Results

### High-throughput screening (HTS) platform for discovering candidate drugs as alternatives to MMC

To identify novel candidate adjuvants for trabeculectomy, we used cell-based HTS assays and drug screening. This method included three assays: (i) a proliferation assay to select drugs that had a stronger suppressive effect on mTCFs than MMC; (ii) an assay to exclude drugs that were toxic to human corneal epithelial cells; and (iii) a flow cytometric analysis to identify drugs that could induce apoptosis in mTCFs. Our aim was to discover novel, clinically useful drugs as alternatives to MMC. This led us to use fibroblasts derived from the ocular tissue of the common marmoset (*Callithrix jacchus*) because of its close genetic relationship with humans^16^.

**Figure 1:**
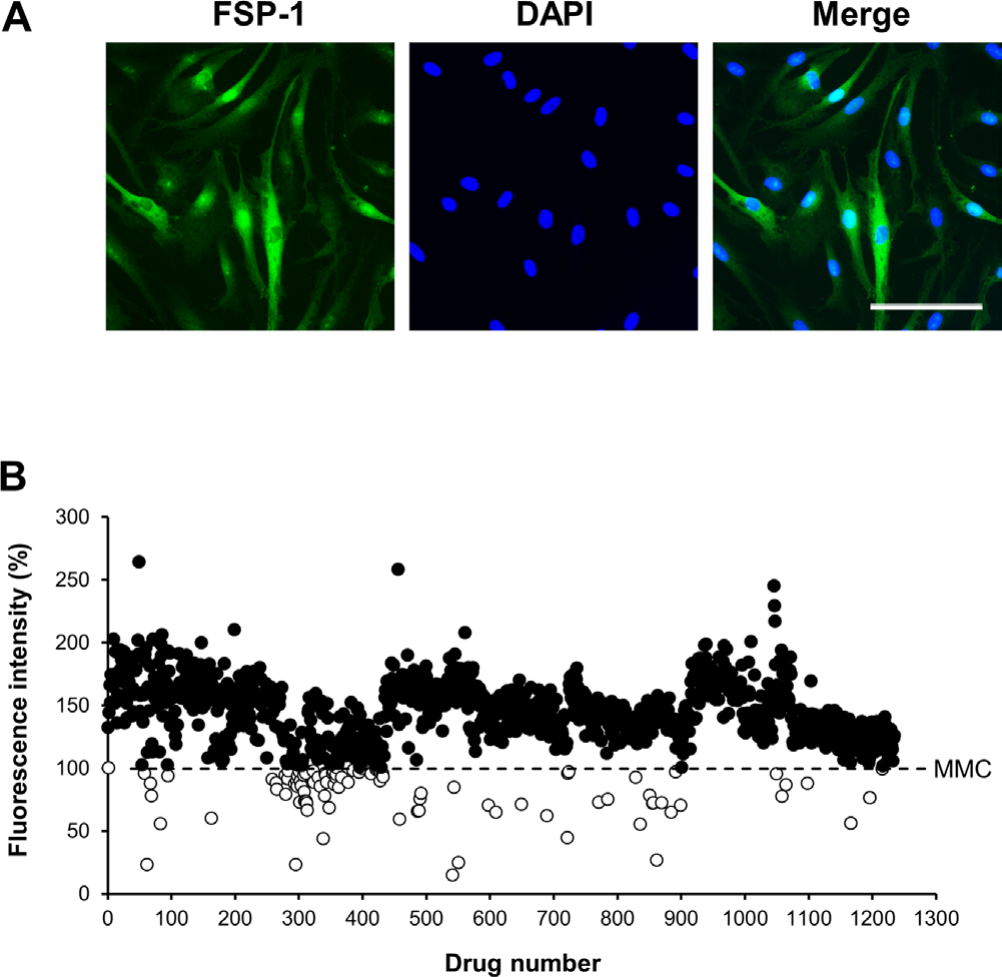
Primary screening of the candidate compounds in mTCFs. (**A**) Characterization of mTCFs with fibroblast-specific protein-1 immunostaining. The scale bar is 100 μm. (**B**) Scatter plot showing results of the primary screening to identify compounds able to inhibit cell proliferation, with an Alamar Blue assay. Ten μM MMC was used as a positive control. The fluorescence intensity after MMC treatment was normalized as 100% cell proliferation. Compounds were selected as candidates (shown by the white circles, 90 total) if they exerted an equal or stronger suppressive effect on fibroblast proliferation compared to MMC. The candidate compounds are all located below the dashed line.

Firstly, we validated our HTS system for screening mTCFs, which we characterized according to their FSP-1 expression (Fig. 1A). We determined the Z-factor with a previously reported formula (Scheme 1)^17^. The Z-factor is generally used as a statistical parameter for evaluating and validating HTS systems. To calculate the Z-factor, we used 0.1% dimethylsulfoxide (DMSO) as a negative control and 10 μM MMC as a positive control. We determined the Z-factor to be 0.63 with these controls, a result that can be considered excellent. Primary screening with this HTS system was performed on 1,165 off-patent drugs (Supplementary Dataset). We found that 90 compounds exerted an equal or stronger suppressive effect on mTCF proliferation than 10 μM MMC (Fig. 1B).

$${\rm{Z}}-{\rm{factor}}=1-\frac{3\times ({\rm{SD}}\,{\rm{of}}\,{\rm{positive}}-{\rm{SD}}\,{\rm{of}}\,{\rm{negative}})}{|{\rm{mean}}\,{\rm{of}}\,{\rm{positive}}-{\rm{mean}}\,{\rm{of}}\,{\rm{negative}}|}$$

**Scheme 1.** Equation of state for Z-factor calculations. SD, standard deviation.

### Cytotoxicity of initial candidate compounds in human corneal epithelial cells

**Figure 2:**
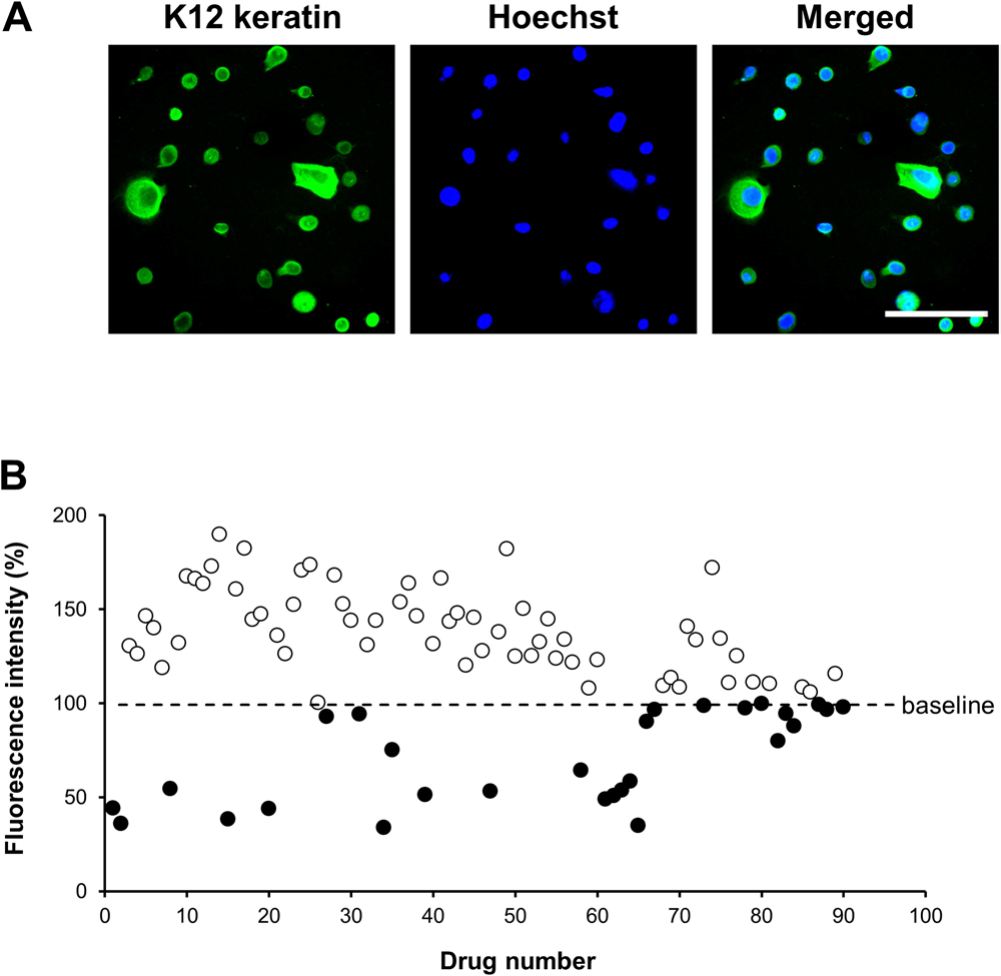
Second screening of the candidate compounds in corneal epithelial cells. (**A**) Characterization of human corneal epithelial cells with cornea-specific keratin K12 immunostaining. The scale bar is 100 μm. (**B**) Scatter plot showing results of the secondary screening to detect cell toxicity, with an Alamar Blue assay. The fluorescence intensity after DMSO treatment was normalized as 100% cell proliferation. Candidate compounds (shown by the white circles, 61 total) were selected from among those showing no cytotoxicity against human corneal epithelial cells.

Corneal epitheliopathy is well known as a complication of trabeculectomy with MMC^18,19^. Then, we used a primary culture of human corneal epithelial cells to evaluate the cytotoxicity of the candidates. Immunocytochemistry for keratin K12, a specific corneal epithelial cell marker, showed that anti-keratin K12 immunoreactivity was present in all cells (Fig. 2A). We identified 61 compounds (including MMC) that did not show cytotoxicity in human corneal epithelial cells from among 90 candidates selected in the primary screening study (Fig. 2B). A lack of cytotoxicity was defined as cytotoxicity lower than that of DMSO as vehicle.

### Identification of compounds that induced apoptosis in the mTCFs

Stronger apoptosis induction is favorable to maintain the bleb’s function, because fibroblast apoptosis reduces fibrosis in tissues^4^. Thus, we performed flow cytometric detection of phosphatidylserine on the outside of the cell membranes with Annexin V, in order to investigate apoptosis-related phenomena. We also performed a cell proliferation assay with the Alamar Blue reagent.

**Figure 3:**
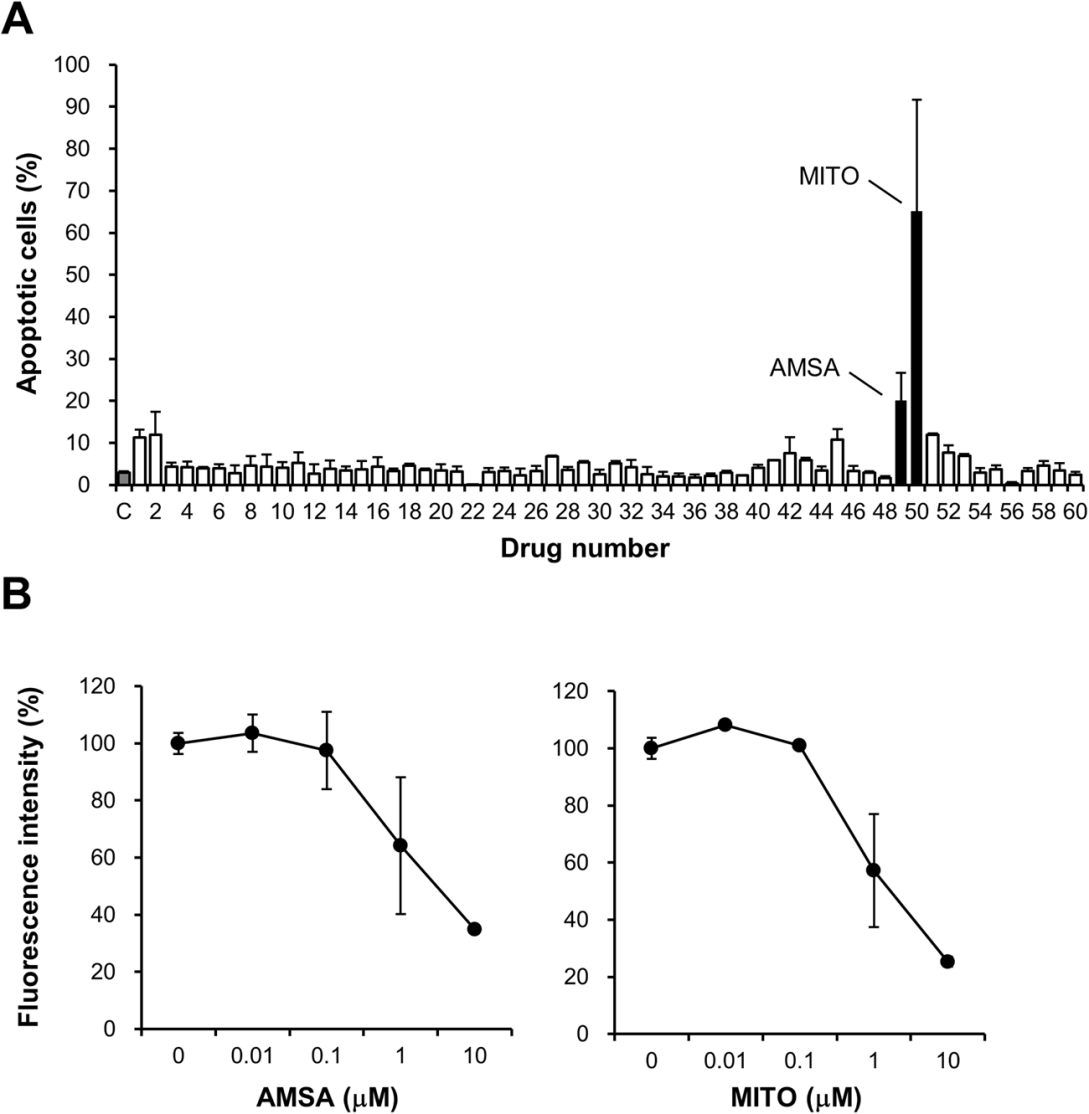
Third screening of the candidate compounds in mTCFs. (**A**) Flow cytometric analysis of apoptosis in mTCFs treated with 60 candidate compounds. This analysis included only 60 candidates, because the 61 candidate compounds identified in the secondary screening included MMC, which was removed. The cells were analyzed with flow cytometry for Annexin V-FITC and 7-AAD fluorescence to determine the number of apoptotic and necrotic cells. The data are shown as the number of fluorescent cells per 340,000 cells after treatment with each compound (n = 2). The DMSO-treated control group is shown as C with a gray bar, while the AMSA and MITO groups are shown with black bars. (**B**) The inhibition of mTCF proliferation after treatment with AMSA and MITO was dose-dependent. Twenty-four hours after treatment with these compounds (0.01–10 μM), a cell proliferation assay was performed with the Alamar Blue reagent (n = 2). The fluorescence intensity of the mTCFs after DMSO treatment was normalized as 100% cell proliferation. Error bar: standard deviation.

Firstly, we examined 60 candidate compounds (selected in the secondary screening study without MMC) in the flow cytometric analysis (Fig. 3A). We identified AMSA and mitoxantrone (MITO) as compounds that were apparent apoptosis inducers (i.e., DNA topoisomerase II (TOP2) inhibitors). The percentages of apoptotic cells stained with Annexin V-FITC in the DMSO (control), AMSA and MITO groups were 3.0% ± 0.3, 20.1% ± 6.6 and 65.2% ± 26.4, respectively. Twenty-four hours after treatment with each drug, the proportion of 7-amino actinomycin D (7-AAD)-positive cells, including late apoptotic, necrotic and dead cells, was negligible (<1.2%).

Finally, we used an Alamar Blue assay to examine the reduction in cell proliferation after treatment with AMSA or MITO. This assay confirmed that both compounds had a dose-dependent effect (Fig. 3B).

### Draize ocular irritation profiles for AMSA and MITO

To find the maximum safe concentrations of AMSA and MITO, we evaluated ocular irritation after treatment with 0.1–10% AMSA and 0.1–10% MITO. These drugs were applied to the bulbar conjunctiva for 5 min. Ocular irritation was similar in the eyes treated with 0.1–10% AMSA and the contralateral eyes treated with saline, with scores of 0.0 + 0.0 at all observed time points. In the eyes that received 0.1–10% MITO, the palpebral conjunctiva was red and the conjunctiva showed chemosis 24 h after treatment, with scores in the 0.1%, 1%, and 10% MITO groups of 0.7 + 0.7, 3.3 + 0.7, and 3.3 + 0.7 (n = 3, mean ± S.E.M), respectively.

These results showed that AMSA led to fewer complications than MITO, and we therefore used AMSA in all following experiments.

### Changes in IOP after treatment in a rabbit model of trabeculectomy

The mean initial IOP value in all groups was between 19.3 mmHg and 19.5 mmHg. The mean initial difference in IOP in the treated and untreated eyes in all groups was between −0.9 mmHg and −0.4 mmHg. No statistically significant differences were observed in inter-animal or intra-eye initial IOP differences. The animals showed no significant ocular or general abnormalities during the experiment.

**Figure 4:**
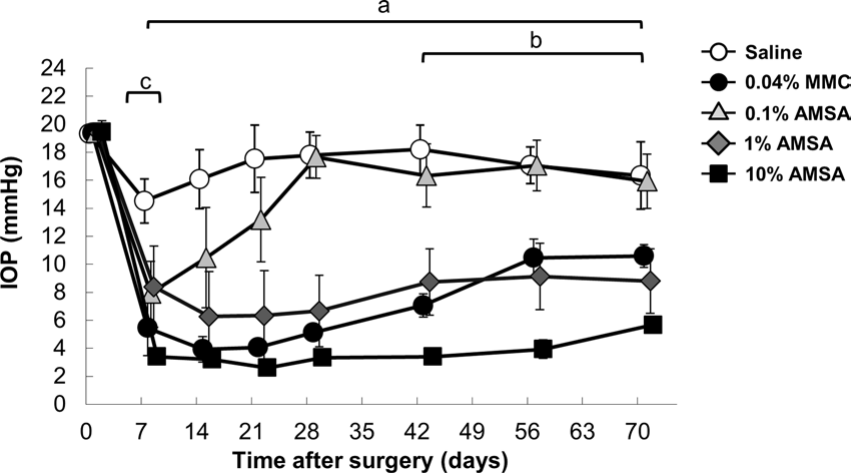
Changes in IOP of after trabeculectomy. Changes in IOP after surgery with saline, 0.04% MMC, and 0.1–10% AMSA. The white circles, black circles, light gray triangles, dark gray diamonds and black squares indicate saline, 0.04% MMC, 0.1% AMSA, 1% AMSA, and 10% AMSA, respectively (n = 5 in each group). The error bar indicates the standard error of the mean. Differences among these groups were analyzed with a two-way repeated ANOVA with Bonferroni post-hoc test (*P* < 0.05 was considered to be statistically significant). Bars a, b and c indicate time periods with a significant difference between groups. Bar a: 10% AMSA, 1% AMSA, and 0.04% MMC vs. saline. Bar b: 10% AMSA vs. 0.04% MMC. Bar c: 1% AMSA vs. 0.04% MMC.

IOP at each measured time point in the five groups is shown in Fig. 4. Compared to saline, IOP decreased significantly in the 0.04% (0.4 mg/ml) MMC, 1% AMSA, and 10% AMSA groups between 7–70 days (all time points: *P* < 0.01). The 10% AMSA group showed significant reductions in IOP vs. MMC at the 42–70-day time points (all time points: *P* < 0.01). The MMC group showed significant reductions in IOP vs. 1% AMSA at the 7-day time point (*P* = 0.02). We also found that 0.1~10% AMSA showed stronger IOP reductions on dose-dependent manner.

### Morphology and bleb scores in rabbit eyes

**Figure 5:**
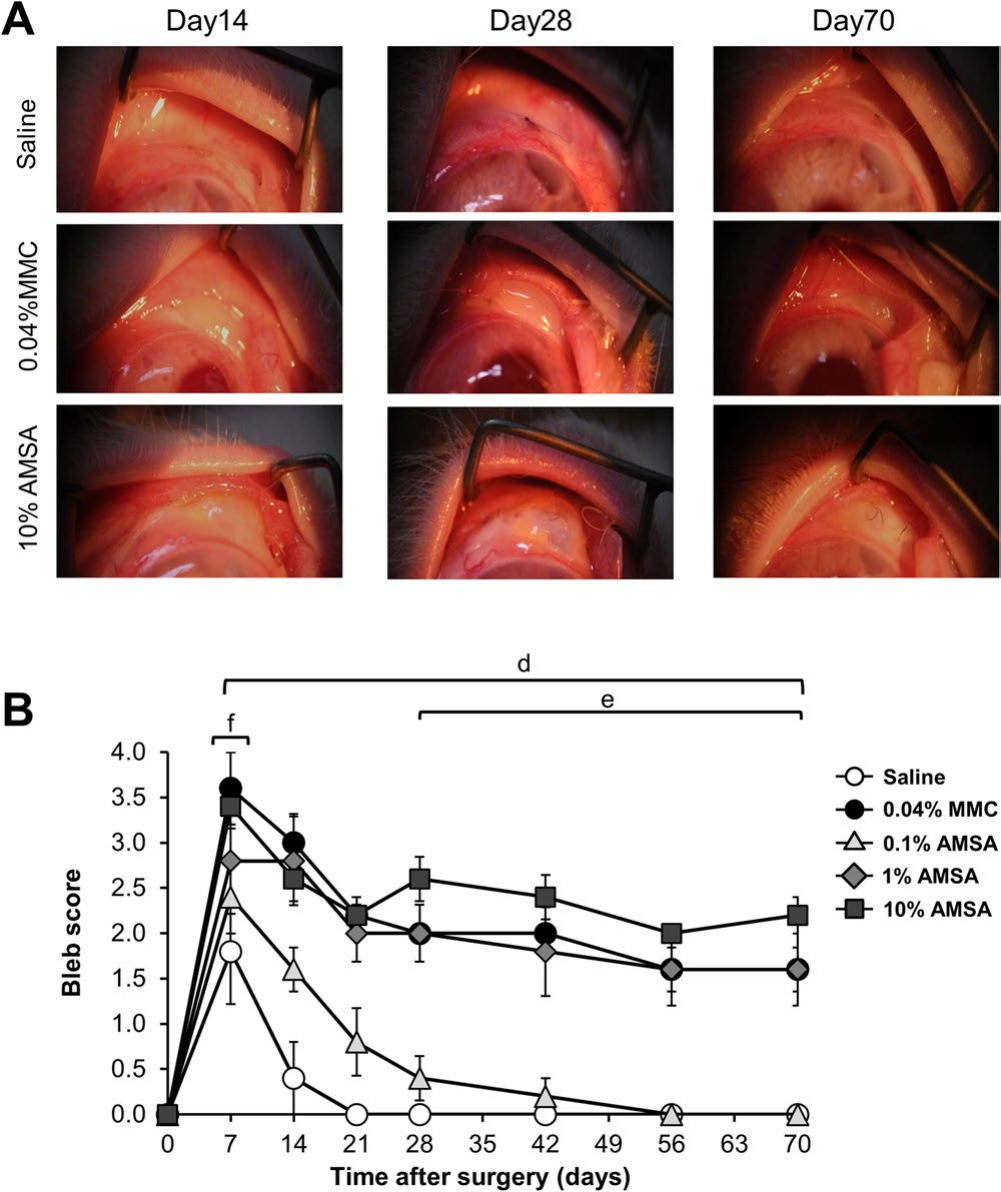
Representative photographs of the blebs and changes in bleb score after trabeculectomy. (**A**) Photographs of blebs after treatment with saline, 0.04% MMC, or 10% AMSA 14, 28 and 70 days after surgery. The blebs are visible in the upper right of each image. (**B**) Changes in bleb score after surgery with saline, 0.04% MMC, and 0.1–10% AMSA. The white circles, black circles, light gray triangles, dark gray diamonds and black squares indicate saline, 0.04% MMC, 0.1% AMSA, 1% AMSA, and 10% AMSA, respectively (n = 5 in each group). The differences among these groups were analyzed with the two-way repeated ANOVA with Bonferroni post-hoc test (*P* < 0.05 was considered to be statistically significant). The error bar indicates the standard error of the mean. Bars d, e, and f indicate time periods with a significant difference between groups. Bar d: 10% AMSA, 1% AMSA, and 0.04% MMC vs. saline. Bar e: 10% AMSA vs. 0.04% MMC. Bar f: 1% AMSA vs. 0.04% MMC.

This study used the method described by Perkins TW to assign a qualitative score for each bleb, representing the size and morphological appearance of the bleb^20^. Figure 5A shows images of blebs in the saline, 0.04% MMC, and 10% AMSA groups. Figure 5B shows average bleb scores in the five groups at each time point. Bleb score was higher in the 0.04% MMC, 1% AMSA, and 10% AMSA groups at each time point between 7–70 days (all time points: *P* < 0.01) compared to saline. The 10% AMSA group had a significantly higher bleb score than the MMC group at 28–70 days (*P* = < 0.01, 0.04, 0.04, and <0.01, respectively). The MMC group had a significantly higher bleb score than the 1% AMSA group at 7 days (*P* < 0.01). We also found that treatment with 0.1~10% concentrations AMSA improved bleb score in a dose-dependent manner.

## Discussion

This study searched for novel alternatives to MMC as adjuvants to trabeculectomy. We screened a library of off-patent drugs with an HTS system based on primary cultured marmoset fibroblasts and human corneal epithelial cells, and identified AMSA, a TOP2 inhibitor, as a promising candidate adjuvant.

Our results demonstrated that compared to saline, AMSA during trabeculectomy led to greater IOP reduction and improved bleb morphology, and compared to MMC, 10% AMSA maintained lowered IOP for a longer period. In fact, IOP in a group of animals that received 0.04% MMC rapidly increased after 28 days, while IOP in a group of animals that received 10% AMSA increased only very slowly after 28 days; IOP in animals that received 10% AMSA was thus significantly lower than the 0.04% MMC animals between 42–70 days. Similarly, the morphology of the bleb after treatment with 10% AMSA was generally more favorable than after treatment with 0.04% MMC after 28 days. An additional interesting finding of this study was that AMSA at a lower concentration of 1% was effective in lowering IOP, but was milder than either MMC or 10% AMSA.

These results indicate that 10% AMSA may be useful for improving the long-term survival of filtering blebs and that 1% AMSA may be a safe option for lowering IOP during the early postoperative period after trabeculectomy. Our findings thus suggest that AMSA is a promising option for bleb preservation after trabeculectomy.

TOP2 has crucial functions, including DNA replication^21^, and is widely known as a clinically important target for chemotherapy in cancer^22,23^. TOP2 has recently attracted interest as a potential target for therapy to prevent bleb failure after glaucoma surgery^24,25^. A previous study reported that daunorubicin, a TOP2 inhibitor, maintained IOP at a low level after trabeculectomy in human patients and contributed to an increased surgical success rate, without corneal epithelial toxicity or other serious complications^25^. However, there have been no reports providing conclusive evidence on the long-term effectiveness or safety profile of TOP2 inhibitors in comparison with MMC. Therefore, our finding that the use of AMSA led to a better prognosis for the filtering bleb than MMC is important, and makes this the first report to show that compounds superior to MMC may be a useful part of clinical care.

The wound healing cascade is divided into four phases: hemostasis, inflammation, proliferation and remodeling^26^. Unlike in humans, the proliferative phase of wound healing in rabbits peaks within the first 14 days^27^. Our results show that postoperative IOP in rabbit eyes treated with 10% AMSA was lower than in eyes treated with saline on day 7 after surgery. This suggests that AMSA is effective and begins to act during an early phase of wound healing. Furthermore, we found that the difference in postoperative IOP between the 0.04% MMC and 10% AMSA groups started to widen after 21 days. This suggests that AMSA might also have an additional mechanism of action during the remodeling phase, which in rabbits corresponds to the period later than day 21 after surgery. We speculate that this is because AMSA might target the matrix metalloproteinases (MMPs), which are well known to have an important role in the remodeling phase^28^. Successful remodeling after trabeculectomy is important for maintaining good bleb function, in addition to suppressing fibroblast proliferation. Indeed, inhibiting TGF-β signaling has been proposed as a therapy targeting the remodeling phase of wound healing^11,12^. TGF-β is proteolytically activated by MMPs and facilitates extracellular matrix remodeling after surgery^29,30^. Thus, suppressing MMPs is also a potential therapeutic target. Indeed, the subconjunctival injection of MMP inhibitors postoperatively significantly improves surgical outcomes and reduces tissue damage^31,32^. AMSA is known to suppress MMP-2 and MMP-9 expression in human leukemia cells via reactive oxygen species (ROS) generation^33^. In this study, the candidate compounds included various TOP2 inhibitors, but AMSA was the only member of the class of acridine-based compounds, which play an essential role in ROS generation^34^. By contrast, MMC does not suppress the activity of MMPs in human Tenon’s fibroblasts^35^.

In this study, we found that after treatment with 10% AMSA the eyes exhibited neither corneal damage nor obvious ocular irritation in a Draize test. Moreover, our results demonstrated that the 10% AMSA group had strong IOP reduction without corneal epitheliopathy for 70 days. This suggests that the clinical use of AMSA during trabeculectomy should not impose a greater risk of corneal epitheliopathy than MMC, even if the Draize test does not directly evaluate post-surgical bleb complications. The finding that AMSA did not lead to corneal epitheliopathy, despite a much higher dose implies that it has a different mechanism of action. This may be explained by the fact that MMC is an alkylating agent, while AMSA is a TOP2 inhibitor. MMC has a cytotoxic effect and inhibits cell proliferation with a bi-directional mechanism. It not only halts the broad-phase cell cycle (i.e., the S, G2/M phase and the G2 phase) but also independently induces cell apoptosis during the cell cycle^36,37^. A previous report has showed that MMC is cytotoxic in corneal tissue, acting via cellular apoptosis^38^. MMC induces cell death in various phases, in both dividing cells and stationary-phase cells. Thus, MMC is a potent inhibitor of cell proliferation regardless of the stage or speed of cell growth. By contrast, TOP2 inhibitors such as AMSA are cytotoxic in a cell cycle-dependent manner, e.g., only during certain phases^39^. Thus, the effect of these compounds may vary in different ocular tissues based on the duration of the cell cycle. For example, fibroblasts have a shorter duration of the cell cycle than corneal epithelial stem cells: 9–15 hours vs. about 72 hours, respectively^40,41^. Therefore, AMSA might selectively affect the Tenon’s fibroblasts and spare the corneal epithelial cells. This might explain why 10% AMSA did not lead to corneal epitheliopathy, despite its much higher dosage. However, AMSA’s mechanism of action leaves open the possibility that it might excessively suppress Tenon’s fibroblasts and, like MMC, cause ischemia in the bleb, with the possibility of various postoperative complications, such as bleb infections, leaks and hypotony^42–44^. To investigate this, we scored the vascularity of the bleb (Supplementary Information)^45^, and found that 10% AMSA led to lower vascularity of the bleb than MMC. Thus, 10% AMSA may be more likely than MMC to cause hypotony and an ischemic bleb, because of its strong apoptotic effect. Interestingly, however, 1% AMSA showed less of a tendency to reduce vascularity in the early postoperative period. This may be the reason why 1% AMSA led to a milder IOP reduction in the early postoperative period and suggests that this dose may be safer and more effective in avoiding hypotony than MMC.

Clinically, our finding that AMSA’s ability to suppress the proliferation of Tenon’s fibroblasts was dose dependent in an animal model suggests that the concentration of AMSA could be varied according to use case. For example, a stronger (10%) dose of AMSA might be suitable for cases of bleb-reconstruction with strongly scarred Tenon’s tissue, in order to prolong IOP reduction, while 1% AMSA might be better for primary cases of trabeculectomy, in order to avoid a sharp drop in IOP just after surgery. Thus, the careful use of AMSA may bring more effective and safer results after trabeculectomy than MMC.

Our study was limited by using a rabbit model of glaucoma filtration surgery. Although this model is well established^46–50^, the animals have normal IOP and are free of glaucoma. Clinical patients often have highly elevated IOP, and the most suitable concentration of AMSA to treat these patients might therefore be very different. Nevertheless, our results may be applicable in normal tension glaucoma (NTG). Various past reports have shown that trabeculectomy to reduce IOP in NTG patients can effectively suppress progression, even when IOP before surgery is within normal limits^44,51^. Thus, trabeculectomy for NTG patients has become an important surgical option in Japan and is widely performed^44,52,53^. Another limitation of our study was that we included no measurements made between 1–7 days after surgery. This leaves open the possibility that surgical variations might have affected the results, although we were able to confirm that there were no perioperative surgical complications, such as hyphema, anastomotic leakage, vitreous prolapse and supra-choroidal hemorrhage.

Another important consideration is the differing affinity of TOP2 inhibitors to two TOP2 isoforms, α and β, which affects their ability to suppress cell proliferation. In mammals, TOP2α and β have different physiological functions and expression patterns^54^. TOP2α affects processes that depend on growth, including the replication of DNA and chromosome segregation^55,56^. TOP2β is not associated with proliferative status and dissociates from the chromosomes during mitosis^54,56,57^. AMSA displays similar activity toward the two isoforms of human TOP2: α and β^58^, while MITO has a stronger effect on TOP2β than TOP2α^59^, causing higher cytotoxicity. This may explain the result of our ocular irritation test, which showed that ocular surface damage occurred after treatment with 1–10% MITO eyes but not with AMSA. In past research, the subconjunctival injection of MITO just after trabeculectomy induced a reduction in IOP, but caused corneal edema in most treated rabbits^60^, while daunorubicin, a potential TOP2α-specific inhibitor, improved the outcome of trabeculectomy in patients with glaucoma without causing serious complications^25^. Thus, TOP2 inhibitors with a low or modest specificity for TOP2β, such as AMSA, may be the most promising candidate adjuvants for trabeculectomy, with the fewest post-operative complications.

In conclusion, we demonstrated the practicality of our HTS system for identifying compounds that are effective. Furthermore, this system identified AMSA as a promising alterative to MMC as an adjuvant to trabeculectomy. The intraoperative use of AMSA during trabeculectomy, at the most suitable concentration for a particular patient, may prolong IOP reduction and be safer than MMC. AMSA is particularly promising because it is already used in clinical practice as an intravenously injected drug. Thus, AMSA might be usable in trabeculectomy without systemic administration.

## Material and methods

### Reagents

In total, 1,165 off-patent drugs from a drug library were provided by Dr. Hideyuki Saya (Keio University, Japan). Dulbecco’s modified eagle’s medium (DMEM), Dulbecco’s phosphate buffered saline (DPBS) and 100 units/ml penicillin/streptomycin were purchased from Life Technologies Inc. (MD, USA). Bovine serum albumin (BSA) was purchased from Sigma-Aldrich (Tokyo, Japan). CnT-20 medium was purchased from CELLnTEC (Bern, Switzerland). Fetal bovine serum (FBS) was purchased from Biological Industries (Beit Haemek, Israel). Amsacrine (AMSA) and (MITO) were purchased from LKT Laboratories, Inc (MN, USA). Kyowa Hakko Kirin Co., Ltd. (Tokyo, Japan) supplied MMC. Daiichi Sankyo Co., Ltd. (Tokyo, Japan) supplied ketamine hydrochloride (Ketalar for intramuscular injection). Bayer Yakuhin Ltd. (Osaka, Japan) supplied xylazine hydrochloride. Shionogi Co., Ltd. (Osaka, Japan) supplied betamethasone sodium phosphate in a 0.01% ophthalmic solution. Santen Pharmaceutical Co., Ltd. (Osaka, Japan) supplied a 0.5% ophthalmic solution of levofloxacin hydrate and a 0.4% ophthalmic solution of oxybuprocaine.

### Primary culture of marmoset Tenon’s capsule fibroblasts (mTCFs)

Enucleated eyeballs of the common marmoset were kindly provided by Dr. Toshio Itoh (Central Institute for Experimental Animals, Japan). The Tenon’s capsule was dissected from the sclera with scissors in DPBS and put onto a 10-cm dish in a minimum volume of DMEM medium containing 1% antibiotics and 10% FBS to avoid floating. Fibroblasts migrated from isolated tissue were incubated at 37 °C for up to 25 days in explant culture. Identification of mTCFs was performed using fibroblast-specific protein-1 (FSP-1) immunostaining.

### Primary culture of human corneal epithelial cells

Primary human corneal epithelial cells were isolated from donor corneoscleral rims provided by Northwest Lions Eye Bank according to a previously described method^61^. Briefly, corneal limbal tissues were incubated in DMEM containing 1.82 units/ml dispase II (Invitrogen, USA) at 37 °C for 1 h. Dissociated human corneal epithelial cells were collected and resuspended in CnT-20 medium with three supplements (Cnt-20.S; CELLnTEC, Switzerland). Identification of human corneal epithelial cells was performed using K12 keratin immunostaining.

### Immunostaining of mTCFs and human corneal epithelial cells

Immunocytochemistry was performed as previously reported^62,63^. Briefly, the mTCFs were fixed in 4% paraformaldehyde (PFA) at room temperature for 10 min, and the human corneal epithelial cells were fixed with cold methanol at −20 °C for 30 min. After washing with DPBS, they were incubated with blocking buffer (10% goat serum, 0.5% gelatin, 3% BSA and 0.2% Tween 20 in DPBS) for 30 min and reacted with primary antibodies against FSP-1 (07–2274; Millipore, USA) or K12 keratin (sc-17098; Santa Cruz Biotechnology, USA) at 4 °C overnight. The cells were washed three times with DPBS containing 0.2% Tween 20 and incubated with an Alexa 488 secondary antibody (Life Technologies Inc., USA) at room temperature for 1 h. FSP-1 and K12 keratin signals were observed using a Zeiss Axiovert 200 microscope (Carl Zeiss, Germany).

### Cell proliferation assay

A drug library comprising 1,165 off-patent drugs was used for identifying inhibitors of the candidate compounds with higher effectiveness than MMC. The mTCFs were placed onto 96-well plates (3,000 cells/mm2) with a Multidrop Combi (Thermo Fisher Scientific, USA) and incubated at 37 °C overnight. They were then treated with DMEM containing the candidate drugs at a final concentration of 10 μM. Twenty-four hours later, the cells were incubated in DMEM containing 10% Alamar Blue reagent (Life Technologies Inc., USA) at 37 °C for 2 h in the dark. Fluorescence intensity was measured at 544 nm excitation and 590 nm emission with a Fluoroskan Ascent microplate reader (Thermo Fisher Scientific, USA).

For the evaluation of corneal cytotoxicity, the human corneal epithelial cells were placed onto 96-well plates at 2,000 cells/mm2 and treated with 10 μM mTCF-growth suppressive drugs in CnT-20 medium at 37 °C for 24 h. All following procedures were then performed as above.

### Analysis of apoptotic cells with flow cytometry

The mTCFs placed onto 12-well plates (1,000 cells/mm^2^) were treated with selected candidate drugs at a final concentration of 10 μM. Twenty-four hours later, supernatant from each culture and the DPBS used for washing were collected in a tube. Corresponding attached cells from the same well were trypsinized and collected in the same tube. After centrifugation at 900 g for 5 min, the resuspended cells were incubated in binding buffer (10 mM HEPES/NaOH, pH7.4, 140 mM NaCl, 2.5 mM CaCl) containing 2.5% Annexin V-FITC (Enzo Life Sciences, USA) and 0.5% 7-aminoactinomycin D (7-AAD; Life Technologies Inc., USA). Ten minutes after incubation in the dark, the cells were washed once with binding buffer and the resuspended cells in the same buffer were analyzed with a BD FACS Canto II device (BD Biosciences, USA).

### Animals

Kitayama Labes Co, Ltd., (Nagano, Japan) supplied the study animals: male Japanese albino rabbits (weight: 1.9 to 3.1 kg). The rabbits were kept under a 12-hour light/dark cycle and were given free access to a standard laboratory food and water.

### Ocular irritation test

The ocular irritation studies used a Draize test, as described previously^64^. Eleven male albino rabbits were used in this test, all of which were free of ocular irritation. The rabbits were divided into four test groups. A preparation of the test compound (0.1–10% AMSA or 0.1–10% MITO) or saline was applied to the bulbar conjunctiva of one eye. The test preparation was applied for 5 min with a Medical Quick Absorber pad (Inami Co, Ltd., Japan). After 30 minutes and 1, 2, 4, and 24 hours, a score was assigned to each eye with the Draize method. The Draize method is a weighted sum of six criteria, all of which are observed directly in the ocular anterior: density, corneal opacification, iritis, conjunctival redness, edema, and discharge (A, B, C, D, and E, respectively, scored at 1–4, 1–4, 1–2, 1–3, 1–4, and 1–3, respectively). Total score was calculated with the following formula: (A × B × 5) + (C × 5) + (D + E + F) × 2; the mean score thus ranged from 0 to 110.

### Glaucoma filtration surgery in rabbits

The rabbits were assigned to one of five groups (saline group: five rabbits, MMC group: five rabbits, 0.1% AMSA group: five rabbits, 1% AMSA group: five rabbits, 10% AMSA group: five rabbits). General anesthesia was induced with intramuscular ketamine hydrochloride (40 mg/kg) and xylazine hydrochloride (4 mg/kg). Next, trabeculectomy was used to create a filtering bleb, using previously described techniques^49,50^. A conjunctival flap was first created in the limbus, after which a scleral flap was created. A Medical Quick Absorber pad soaked in saline, 0.04% MMC, or 0.1–10% AMSA was placed on the scleral flap under the conjunctiva for 5 min. The area was then irrigated with sodium chloride solution (120 ml, 0.9% concentration; Otsuka, Japan). Next a sclerotomy was created under the flap with a Kelly Descemet’s membrane punch (M.E. Technica, Japan) being used to create a scleral tunnel into the aqueous chamber. The conjunctiva was then sutured with 10–0 sutures; he scleral flap was not sutured. Topical administrations of 0.01% betamethasone sodium phosphate and 0.5% levofloxacin hydrate ophthalmic solution were then applied four times a day for five days after surgery. No perioperative complications, such as hyphema, anastomotic leakage, vitreous prolapse and supra-choroidal hemorrhage, were observed in any of the eyes. In all groups, IOP was measured and the bleb was evaluated 7, 14, 21, 28, 42, 56 and 70 days postoperatively.

### Measurement of IOP

A pneumatonograph (Model 30 Classic Pneumatonometer; Reichert Technologies, USA) was used to measure IOP after general anesthesia was induced with intramuscular ketamine (40 mg/kg) and xylazine (4 mg/kg). IOP was measured in both eyes 7 minutes after the anesthetic was injected. Corneal anesthesia was induced with topical 0.4% oxybuprocaine before IOP measurement. All IOP measurements were performed after 3 pm and before 7 pm in a normal laboratory environment with stable lighting.

### Bleb evaluation

Bleb examination used a slit lamp (Portable Slit lamp SL-15; Kowa Company. Ltd., Japan). The blebs were graded qualitatively, using a modification of Perkins’s method^18^. The blebs were graded from 0 to 4+. A higher score indicated greater bleb size and height: 0 represented an absent bleb; 1+ represented a short bleb with conjunctival thickening and no microcysts; 2+, represented a bleb with microcysts covering less than 75° of the eye; 3+ represented a high bleb with microcysts covering 75 to 135°; and 4+ represented a very high bleb with microcysts covering more than 135°.

### Statistical analysis

Firstly, we compared IOP between treated and contralateral untreated eyes before treatment in five groups: saline, 0.04% MMC, 0.1% AMSA, 1% AMSA, and 10% AMSA. These comparisons used the Tukey-Kramer test for multiple comparisons (statistical significance was set at *P* < 0.05).

Next, to confirm the effect of MMC and AMSA, we compared treated-eye IOP in the five groups. This comparison used a two-way repeated ANOVA with Bonferroni post-hoc test (statistical significance was set at *P* < 0.05). Bleb morphology and bleb score were compared in the five groups with a two-way repeated ANOVA with Bonferroni post-hoc test (statistical significance was set at *P* < 0.05).

### Ethics statement

All experiments including the human cell experiments were approved by the institutional review board of Tohoku University Graduate School of Medicine (Approval number: 2014-2-98-1), and followed the tenets of the Declaration of Helsinki.

This study conformed to the ARVO Statement for the Use of Animals in Ophthalmic and Vision Research. Additionally, the study was approved by the Animal Care and Use Committee of Santen Pharmaceutical Co., Ltd (Approval numbers: DR130356 and DR-2014-0035). An intravenous injection pentobarbital was used to euthanize the animals in all cases.

## Supplementary information

Supplementary Figure 1. Bleb vascularity score

Dataset 1

## Data Availability

The datasets generated during and/or analyzed during the current study are available from the corresponding author on reasonable request.
