# Supplementary material for: The DNA topoisomerase II inhibitor amsacrine as a novel candidate adjuvant in a model of glaucoma filtration surgery
Source: Sci Rep. 2019 Dec 17;9:19288. doi: 10.1038/s41598-019-55365-7 (PMC6917768; doi:10.1038/s41598-019-55365-7)
Supplement: Supplementary file 1 — Supplementary Figure 1. Bleb vascularity score [file 41598_2019_55365_MOESM1_ESM.pdf]

<Title>

The DNA topoisomerase II inhibitor amsacrine as a novel candidate adjuvant in a model of glaucoma filtration surgery

Supplementary information

<Authors>

Kotaro Yamamoto*[1], Taiki Kokubun*[1], Kota Sato[1, 2], Takahiro Akaishi[3], Atsushi Shimazaki[3], Masatsugu Nakamura[3], Yukihiro Shiga[1], Satoru Tsuda[1], Kazuko Omodaka[1], Hideyuki Saya[4], Toru Nakazawa[1, 2, 5, 6, 7]

*KY and TK contributed equally to this work.

Supplementary Figure 1. Bleb vascularity score

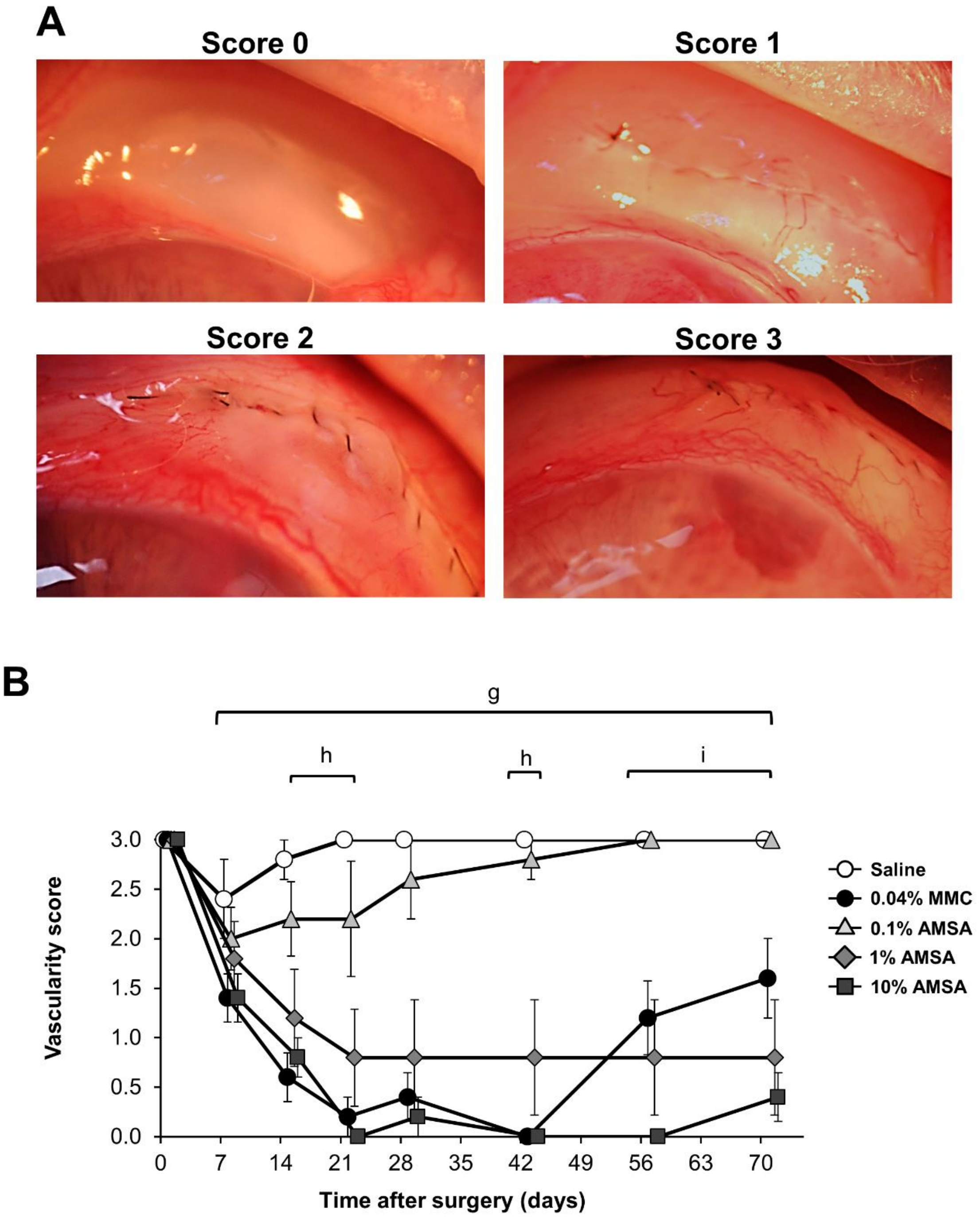

(A) Representative images showing vascularity score (0-3) according to Wuerzburg's method (reference 45). Briefly, with increasing score, vessels cover a wider area of the bleb.

(B) Changes in vascularity score after surgery with different adjuvants. The white circles, black circles, light gray triangles, dark gray diamonds and black squares indicate saline, 0.04% MMC, 0.1% AMSA, 1% AMSA, and 10% AMSA, respectively (n = 5 in each group). The treatment groups were compared with a two-way repeated ANOVA with Bonferroni post-hoc test (statistical significance: $P < 0.05$). The error bar indicates the standard error of the mean. Bars g, h, and i indicate time periods with a significant difference between groups. Bar g: 0.04% saline vs. 1% AMSA, 10% AMSA, or 0.04% MMC. Bar h: 1% AMSA vs. 0.04% MMC. Bar i: 10% AMSA vs. 0.04% MMC.
